# Electrophysiological Comparison of Cumulative Area and Non-Symbolic Number Judgments

**DOI:** 10.3390/brainsci13060975

**Published:** 2023-06-20

**Authors:** Justin W. Bonny, Stella F. Lourenco

**Affiliations:** 1 Department of Psychology, Morgan State University, 1700 East Cold Spring Lane, Baltimore, MD 21251, USA; 2 Department of Psychology, Emory University, Atlanta, GA 30332, USA; stella.lourenco@emory.edu

**Keywords:** approximate number system, magnitude representation, congruity effect, ratio effect, ERP, non-symbolic number judgments

## Abstract

Despite the importance of representing different magnitudes (i.e., number and cumulative area) for action planning and formal mathematics, there is much debate about the nature of these representations, particularly the extent to which magnitudes interact in the mind and brain. Early interaction views suggest that there are shared perceptual processes that form overlapping magnitude representations. However, late interaction views hold that representations of different magnitudes remain distinct, interacting only when preparing a motor response. The present study sheds light on this debate by examining the temporal onset of ratio and congruity effects as participants made ordinal judgments about number and cumulative area. Event-related potentials (ERPs) were recorded to identify whether the onset of such effects aligned with early versus late views. Ratio effects for both magnitudes were observed starting in the P100. Moreover, a congruity effect emerged within the P100. That interactions were observed early in processing, at the same time that initial ratio effects occurred, suggests that number and cumulative area processes interacted when magnitude representations were being formed, prior to preparing a decision response. Our findings are consistent with an early interaction view of magnitude processing, in which number and cumulative area may rely on shared perceptual mechanisms.

## 1. Introduction

Making decisions about the relative differences in quantities, such as the sizes of vegetables or the numbers of people in grocery store checkout lines, without the use of symbolic tools (e.g., rulers or counting), relies in part on our ability to represent mental magnitude. Referred to as magnitude representations, these are mental estimates of non-symbolic magnitudes constructed from perceptual information. Accumulating evidence suggests that magnitude representations are involved not just in making basic decisions about relative differences, but may also be connected to mathematical skills [1,2,3,4,5,6]. Moreover, we typically consider multiple types of magnitudes when planning and performing actions [7,8]. For example, cumulative area (i.e., the total area occupied by objects within a set) and number are both relevant when planning how many boxes will fit into a vehicle. Though magnitude representations influence decision-making and action planning [9,10], questions remain as to whether, and how, different magnitudes such as cumulative area and number are simultaneously encoded. Event-related potentials (ERPs) have previously been used to identify the cognitive processes underlying magnitude representations during Stroop-like comparison tasks [11,12,13]. In the present research, ERPs were recorded during a magnitude comparison task to examine whether the emergence of cumulative area representations is affected by non-symbolic number representations, and vice versa, with the goal of informing models of magnitude processing.

### 1.1. Approximate Magnitude Representations: Number and Cumulative Area

Whereas number corresponds to the discrete quantity of a set of objects (e.g., how many dots are in an array), cumulative area refers to the continuous spatial extent occupied by a set of objects (e.g., how many pixels the array of dots encompasses). Number and cumulative area are ideal to examine the underlying cognitive mechanisms of magnitude representation as both are available within the same visual scene. This can lead to situations where both magnitudes may or may not be congruous with one another. For example, in a classroom full of adults and children, there could be more adults in both number and space, or there could be more children in number, but the physically larger adults take up more space than the physically smaller children. Since each magnitude can point to a different response if judging which is greater (in the latter example: number points to children, cumulative area points to adults), the encoding processes for each magnitude are typically considered distinct from one other [14,15,16,17]. Two effects in behavioral and neural performance have been used to compare processes involved in number and cumulative area judgments: the ratio and congruity effects.

### 1.2. Ratio and Congruity Effects

The ratio effect in behavior refers to greater performance (e.g., higher accuracy, faster reaction times) as the ratio between two stimuli increases (i.e., larger ratio, when the larger value is divided by the smaller value), in line with Weber’s law. The ratio effect suggests the recruitment of approximate magnitude representations and has been used to evaluate the emergence of non-symbolic magnitude representations (e.g., [18,19,20]). The putative cause of the ratio effect is the inherently approximate nature of magnitude representations, modeled as they are on a Gaussian distribution placed upon a mental “more versus less” continuum [21,22]. Comparison tasks, in which participants judge which of two non-symbolic magnitude values is greater, have previously been found to elicit ratio effects for both cumulative area and non-symbolic number judgments [23,24], making such tasks ideal for examining the nature of the underlying magnitude representations. Ratio effects have also been observed in neural activity. In fMRI research, BOLD activity in the intraparietal sulcus (IPS) is modulated by the ratio of values for multiple types of non-symbolic magnitudes [25,26,27,28,29]. Ratio effects for different non-symbolic magnitudes observed in behavioral and neural data have led to proposals of common underlying cognitive mechanisms [7,30,31,32]. However, questions remain about the emergence of magnitude representations and whether overlap occurs during early or late processing. Tracking the onset of the formation of these representations is essential in distinguishing the nature of the overlap between magnitudes.

Congruity effects are identified when performance during a task is affected by the congruency of dimensions within a stimulus. In comparison tasks with two types of magnitudes present in the stimuli (e.g., number and area), the relation between magnitudes affects performance. When the magnitudes are congruent—they are both in the same direction (e.g., the stimulus that is larger in number is also larger in area)—performance is generally high. When the magnitudes are incongruent–they are in different directions (e.g., the stimulus that is larger in number is smaller in area)—performance is generally low. Congruity effects have been observed with tasks using Arabic numerals of different sizes (e.g., _2_ and 8 vs. 2 and _8_) both behaviorally [33,34,35] and in neural activity, particularly within the IPS and frontal regions [13,36,37]. Congruity effects have also been observed with non-symbolic stimuli (e.g., arrays of dots; behavioral [11,38,39]). Together, ratio and congruity effects have been used to investigate the precision of representations and interactions during magnitude processing.

### 1.3. Timing of Interaction Effects in Non-Symbolic Magnitude Tasks

Using ERPs, which have high temporal precision, the earliest point at which a ratio effect emerges suggests the initial formation of a magnitude representation [40,41]. Non-symbolic number ratio effects typically emerge as a positive deflection from baseline processing between 180 and 250 ms after stimulus onset, also known as the P200. Modulation of the P200 ERP waveform has been observed to be most pronounced over lateral posterior sites for non-symbolic numbers, including P1 through P10 and PO3 through PO10 when participants passively viewed stimuli [42] or made comparison judgments [41,43]. When participants were asked to view arrays that varied in one of multiple non-symbolic magnitudes, including cumulative area and number, without making an explicit magnitude judgment, ratio effects were observed for cumulative area in N100 (135 to 160 ms) and P200 waveforms over lateral parietal-occipital electrodes, including O1, O2, and PO3 through PO8 [44]. A series of studies investigated the extent to which differences in numerical and non-numerical magnitudes emerged using a rapid serial task where participants viewed arrays differing in more than one magnitude. Differences between numerical and non-numerical magnitudes were observed in the C1 waveform, around 60 through 100 ms after stimulus onset, and as early as 75 ms at the OZ electrode [45]. Furthermore, numerosity-specific patterns varied between the P200 and C1 waveforms [46]. These findings indicate that ratio effects emerge early for numerical and non-numerical magnitudes in visual stimuli. Ratio effects do not, however, provide insight into the nature of potential interactions between magnitudes.

Congruity effects have been used to show that interactions between number and cumulative area occur [10,13,47,48]. However, these effects alone fall short of explaining the nature of the interaction, in particular when in processing it occurs. An early account of congruity effects holds that the interactions between magnitudes occur during perceptual processing, whereas a later account generally argues that interactions occur during the preparation of a motor response when judging, for example, ordinality (i.e., which is greater). The late account holds that magnitude representations remain independent until these later stages, with the interaction occurring when resolving which response to make.

Previous studies using ERPs have suggested that congruity effects in P200 and P300 waveforms reflect an early account of magnitude interactions. By contrast, the presence of congruity effects in the lateralized readiness potential, but not earlier waveforms, has been taken to support a late account. With symbolic stimuli, and when judging which of two digits was greater in number or individual size, the effect was observed in the P200 and P300 waveforms over central and lateral parieto-occipital sites, including PO electrodes [12], earlier relative to later motor response components such as the lateralized readiness potential [12,49]. However, congruity effects with symbolic stimuli have also been observed later than the P300 without corresponding earlier effects, providing evidence that magnitude representations remain independent until interacting when preparing a motor response [12,13,49,50].

In addition to the mixed evidence with symbolic stimuli, there is limited evidence of ERP congruity effects specific to cumulative area when using non-symbolic stimuli. A challenge with using symbolic digits with spatial magnitudes is the interference from the salient semantic content of the numerals. The use of non-symbolic stimuli mitigates such symbolic interference. In a study by Gebuis and colleagues (2010), participants completed two notation conditions where they judged which of two Arabic numerals of different sizes (symbolic condition) or dot arrays (non-symbolic condition) was numerically greater or physically larger. During the task, the absolute difference in the number or physical size was manipulated to be smaller or greater as participants judged which stimulus was either numerically or spatially greater. Although instructions for spatial judgment trials did not specify to participants that they focus on the cumulative area, the design of the stimuli entailed that the cumulative area varied for the non-symbolic condition. A congruity effect for the non-symbolic condition was observed in the P300 over the Pz electrode, favoring the early account [11]. However, results on the physical size task varied from the number task in both notation conditions. Unlike number, the size distance effect for incongruent trials was reversed, with worse performance for large compared to small distances. A reverse pattern for small and large distances was also observed for number and physical size in the latency of the P300 waveform. These differing patterns were discussed as potentially being due to a greater interference of numerical information on spatial judgments [11]. However, the inclusion of symbolic stimuli (Arabic numerals) in addition to non-symbolic dot arrays may have affected the extent to which participants attended to spatial information and minimized cumulative area cues.

Using similar non-symbolic stimuli for both number and cumulative area, and basing spatial judgments specifically on cumulative area, can provide additional ERP evidence of whether interaction effects emerge earlier or later in processing. Furthermore, using ratio and congruity effects together can better inform at what point early interactions are occurring. Addressing when these effects emerge can inform theories of magnitude representation. Evidence of early interactions would suggest that number and cumulative area share processing resources, perhaps in perception, and would run counter to the view that non-symbolic number is represented independently of other magnitudes (e.g., [51,52,53,54]). Proponents of independent number representation argue that congruity effects necessarily reflect late-stage processing. Evidence of effects emerging shortly after magnitude information is presented would provide evidence of, at least partially, overlapping mechanisms during perceptual processing.

### 1.4. The Present Study

In the present study, the onsets of ratio and congruity effects in ERP waveforms for cumulative area and non-symbolic number were compared. During the study, participants made ordinal comparisons, judging which of two non-symbolic arrays was larger in either number or cumulative area. Instructions were selected to emphasize that participants use numerical (number of ‘boxes’) or cumulative area (amount of ‘paint’) to base their judgments for each magnitude. Previous operational definitions of what constitutes an early congruity effect were refined by yoking it to the initial ratio effect. As highlighted by Santens and Verguts (2011), the criteria used to determine whether a congruity effect occurs early in processing have varied widely, ranging from 300 ms to over 600 ms after stimulus onset. Here, ‘early’ was defined as the earliest window in which the ratio effect was observed. Since a ratio effect has been argued to be evidence of a magnitude representation [26,32,40,55], the co-occurrence of congruity with the earliest ratio effect would indicate an early interaction between magnitudes.

We used three different conditions to test for congruity effects (congruent, incongruent, and neutral). In behavioral studies, performance is typically highest for the congruent condition, lowest for the incongruent condition, with the neutral condition falling in between [9,56]. In previous ERP studies, however, the pattern of congruity effects has varied, especially for research using non-symbolic stimuli. The pattern ranges from higher deflections for congruent than incongruent trials to the reverse pattern, and no difference between congruent and incongruent trials [13,48]. The inclusion of a neutral condition was thus important for interpreting similarities or differences between congruent and incongruent conditions. We hypothesized that support for an early interaction effect would be observed. Specifically, initial ratio and congruity effects would emerge within the same ERP waveform for both number and cumulative area judgments.

## 2. Method

### 2.1. Participants

Twenty-four participants recruited from a university campus (19 females, M_age_ = 19.8 years, range = 18.1 to 23.5 years) were included in the final analyses. An additional seven participants were excluded due to excessively low accuracy on the comparison tasks (performance lower than 3 SD), excessive noise in the reference electrodes (3, see below), and a software malfunction (1). Participants were recruited from a university campus using electronic and printed study advertisements. The recruitment criterion was that individuals be willing to have gel applied to their hair as part of study procedures. Participants received either course credit (undergraduate students) or gift cards (staff members) as compensation. All subjects gave their informed consent for inclusion before they participated in the study. The study was conducted in accordance with the Declaration of Helsinki, and the protocol was approved by the Institutional Review Board of Emory University (protocol number IRB00021217 approved in 2011).

### 2.2. Apparatus

The task was presented on a computer monitor (30.5 × 23 cm) and completed using action buttons on a console-style gamepad controller (Logitech, Inc., Newark, NJ) and eevoke software (Advanced Neuro Technology bv, Hengelo, The Netherlands; ANT). Participants were seated approximately 60 cm from the monitor and fitted with an ANT WaveGuard EEG cap with 32 shielded electrodes (Ag/AgCl). The cap was made of lightweight fabric and the electrodes were positioned in a modified International 10–20 system ([57]) and wires were shielded to reduce contamination of the signal from electrical noise from the environment. The electrophysiological signal was recorded using ANT software (version 3.1) running on a desktop computer (Dell, Inc., Round Rock, TX). The signal was amplified 20,000 times and sampled at a rate of 256 Hz. Prior to the test session, ElectroGel (Electro-Cap International, Inc., Eaton, OH) was applied to each electrode to reduce impedances to around or below 15 kΩ.

### 2.3. Stimuli

**Figure 1:**
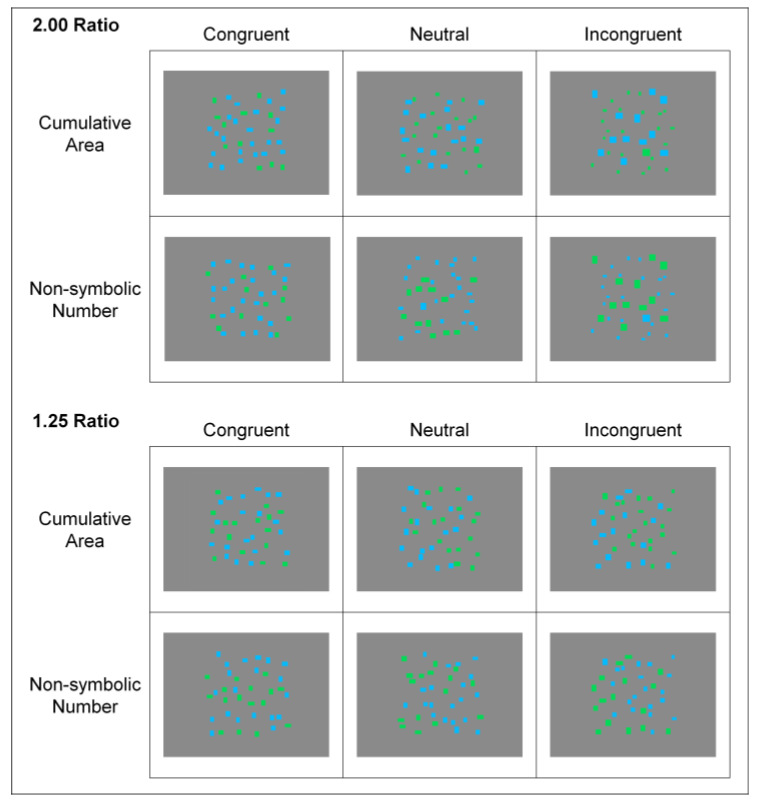
Sample stimuli used for each magnitude by ratio by congruity cell in our magnitude comparison task. The size and number of blue versus green boxes were manipulated to create differences in cumulative area and number, respectively. The samples correspond to trials in which the blue array was greater in the target magnitude.

Images composed of two arrays of blue (RGB color code: 0, 187, 255) and green (rgb color code: 0, 217, 87) rectangles were used to display systematic differences in cumulative area and number. The two arrays were spatially intermixed within a 17.8 × 17.8 cm frame (visual angle of 16.87 degrees) on a gray background (RGB color code: 138, 138, 138); the positions of each element were randomly determined and the color greater in magnitude was counterbalanced across trials (Figure 1).

To create differences in congruity between the target (i.e., cumulative area in the cumulative area condition and number in the number condition) and secondary magnitude (i.e., number in the cumulative area condition and cumulative area in the number condition), the arrays were manipulated to yield three conditions: congruent, neutral, and incongruent. In congruent trials, target and secondary magnitudes were in the same direction (e.g., the array that was larger in cumulative area was also larger in number). In neutral trials, the target magnitude was manipulated between arrays while the secondary magnitude was equated across arrays (e.g., the array that was larger in cumulative area had the same number of rectangles as the other array). In incongruent trials, target and secondary magnitudes were pitted against each other (e.g., the array that was larger in cumulative area was smaller in number). For trials that were congruent or incongruent, the secondary magnitude varied by the same amount as the target magnitude (e.g., for a congruent trial, if the target array was larger in cumulative area by a 2.00 ratio, then it was also larger in number by a 2.00 ratio).

For each congruity condition, the ratio between the cumulative area and the number of rectangles of the blue and green arrays varied according to the particular magnitude that participants were required to judge. The design of the stimuli was based on those used in Lourenco et al. (2012). The ratio was either large (2.00) or small (1.25), similar to what has been used in previous research [40,41,43]. For number, the total number of rectangles of both arrays remained constant across trials (36) but varied between each array according to the ratio (24 vs. 12 for the 2.00 ratio; 20 vs. 16 for the 1.25 ratio). For cumulative area, the total area of both arrays was constant across trials (30.9 cm^2^) but the area of each array varied according to the ratio (20.6 cm^2^ vs. 10.3 cm^2^ for the 2.00 ratio; 17.2 cm^2^ vs. 13.7 cm^2^ for the 1.25 ratio). These parameters were the same across congruent and incongruent conditions. What varied was whether the array that was greater in the target magnitude was also greater in the secondary magnitude. Only in the neutral condition did the values of the secondary magnitude change to maintain a 1.00 ratio (when the number was neutral for the cumulative area condition, 18 rectangles; when the cumulative area was neutral for the number condition, 15.45 cm^2^; additional stimulus details are available in Supplementary Materials; stimuli available via OSF: https://doi.org/10.17605/OSF.IO/RJ7US). The largest rectangle in each array was matched in size to reduce the likelihood that participants would rely on a single rectangle to make their decision. With magnitude, ratio, and congruity conditions, the study was a 2 (magnitude: cumulative area, number) by 2 (ratio: 2.00, 1.25) by 3 (congruity: congruent, neutral, incongruent) design yielding twelve types of trials (see Figure 1).

### 2.4. Procedure

After being fitted with the EEG cap, participants were instructed on how to complete the task. They were told that a series of images would be presented onscreen and that they were to judge whether there was more blue or green “paint” (cumulative area condition) or more blue or green “boxes” (number condition). Researchers confirmed with each participant that they understood the distinction between “paint” and “boxes” before starting the study. To make a response, they pressed either the left or right button on the game controller, which corresponded to either color (each marked with a sticker of the target color; counterbalanced across participants). Participants were told they would be presented with blocks of trials that alternated between making judgments about paint or boxes (order counterbalanced across participants). At the beginning of each block, they were presented with a word prompt that indicated which judgment they were to make (‘PAINT’ for cumulative area condition; ‘BOXES’ for number condition). All prompts were presented in white Arial font (on-screen height: 1.5 cm) on a gray background. For each trial, a fixation point (‘o’ character, Arial font) was presented for a variable amount of time (500 to 1000 ms) after which the stimulus was presented and remained onscreen until the participant made a response. The next trial began immediately following a response. Participants were first presented with four practice trials for each magnitude condition with a highly discriminable ratio (3.00; no feedback given). Afterward, participants were presented with 24 blocks (12 blocks for each magnitude condition) of test trials, each block containing 20 test trials, for a total of 480 test trials. The six trial types for each magnitude (12 total types) were randomly distributed across each set of magnitude-specific test blocks. Unbeknownst to the participant, an additional practice trial (3.00 ratio) was presented at the beginning of each block to minimize the effects of changing instructions; no corrective feedback was provided.

### 2.5. Data Reduction

Behavioral performance on test trials was measured by participants’ accuracy and reaction time (RT). The correct choice of the larger magnitude (1; incorrect = 0) was calculated for each magnitude by ratio by congruity trial. RT was calculated for each correct trial.

**Figure 2:**
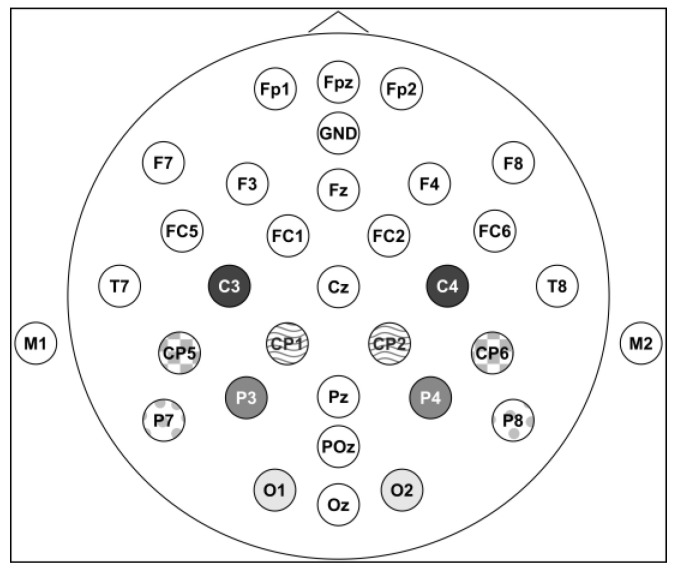
Diagram of electrode placement on ERP cap (including ground) as well as the electrode pairs of focus (dark gray, C3, C4; wavy, CP1, CP2; checkerboard, CP5, CP6; medium gray, P3, P4; dots, P7, P8; light gray, occipital: O1, O2).

ERP data reduction was as follows. On each test trial, the ERP signal from 100 ms prior to stimulus onset until 800 ms after stimulus onset was analyzed (256 Hz rate). Using ASA software (ANT, version 3.1), an offline bandpass filter (frequencies less than 0.1 Hz and more than 30 Hz, 24 dB/octave gain) was applied to reduce environmental artifacts. Further data reduction was completed using the EEGLAB 2021.1 [58] and ERPLAB 9.0.0 [59] toolboxes running in MATLAB R2021b (MathWorks, Natick, MA, USA). Independent component analysis (fast ICA algorithm; [60]) combined with visual inspection was used to identify and remove eye blink components for participants (number of components removed: Median = 1, Min = 1, Max = 2). Afterward, trials that contained amplitudes above 100 μV or below −100 μV were removed to reduce remaining artifacts. All remaining test trials where participants responded correctly were used in statistical analyses (trials per magnitude by ratio by congruity cell: M = 29.11, Median = 32, Min = 2, Max = 40). Participants for which at least one of the mastoid reference channels had excessive noise (consistent fluctuations of 500 μV) were removed from the analyses (N = 3), yielding a total of 24 participants for data analyses. For each cell, the mean amplitude for each waveform component was extracted for each trial and electrode (baseline correction 100 ms prior to the stimulus onset). Twenty-three electrodes were inspected (see Figure 2) after removing electrodes that were either used as references (mastoid references, M1, M2) or contained excessive contamination from muscle artifacts (7: FP1, FP2, FPz, F7, F8, T7, T8). Data analysis included 8384 trials across participants (datasets and analysis script are available via OSF: https://doi.org/10.17605/OSF.IO/RJ7US).

Electrode pairs were selected based on previous research. In previous studies examining the ratio effect, left and right electrode pairs have been created for parieto-occipital and central sites [40,41,43] and studies examining congruity effects have typically focused on electrodes along and near the midline in posterior regions [11,13]. Corresponding electrode pairs were created in the present study for central (C3/C4), central-parietal (CP1/CP2), lateral-central-parietal (CP5/CP6), parietal (P3/P4), lateral-parietal (P7/P8), and occipital (O1/O2) areas (see Figure 2 for positions). In previous research, differences due to format (e.g., digits vs. dot arrays) have been reported in early emerging waveforms, such as the C1 (100–200 ms [46]) and N1 (130–170 ms [43]), and differences due to ratio have been observed in subsequent waveforms, including the P200 (200–500 ms [40,41,43,61]). Congruity effects with non-symbolic stimuli have been observed around 300 ms after stimulus presentation, such as in the P300 [11]. Using this previous research, the following waveforms were identified and analyzed (labeled by amplitude direction and approximate onset after stimulus presentation): P100 (70–140 ms), N100 (141–200 ms), P200 (201–250 ms), and P300 (251–400 ms).

Along with behavioral performance, for each electrode and waveform, the mean amplitude was analyzed in R using the ‘lme4’ [62], ‘lmerTest’ [63], ‘car’ [64], and ‘emmeans’ [65], and visualized using the ‘ggplot2’ [66] libraries. Ratio conditions were contrast coded to align with previous ERP research that observed a greater mean amplitude for smaller ratios (1.25 Ratio = 1; 2.00 Ratio = −1). All statistical tests were two-tailed (α = 0.05) and, for mixed models, degrees of freedom were estimated via Satterthwaite method. Post hoc tests were analyzed for significant main effects and interactions with comparison *p*-values adjusted for family-wise error. For behavioral analyses, post hoc comparison *t*-test *p*-values were adjusted using Tukey HSD. For ERP analyses, *z*-tests were used for post hoc comparisons due to the large number of observations with repeated-measures factors with *p*-values adjusted using Tukey HSD.

## 3. Results

### 3.1. Behavioral Results

**Figure 3:**
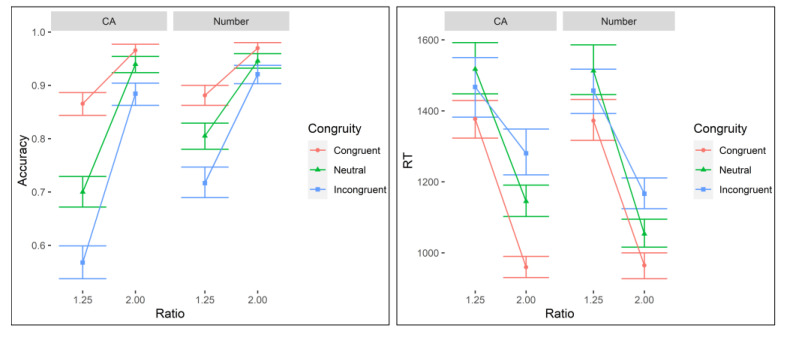
(**Left** panel) Mean accuracy on cumulative area (CA) and non-symbolic number (Number) conditions. Performance was significantly above chance in all cases (*p*s < 0.001). (**right** panel) Reaction times (RT)s for correct trials in the CA and non-symbolic number (Number) conditions. Error bars represent 95% confidence intervals.

A mixed effects logistic regression (binomial link), with magnitude (cumulative area, number), ratio (contrast coded: 1.25 Ratio = 1; 2.00 Ratio = −1), and congruity (congruent, neutral, incongruent) as factors and accuracy as the dependent variable (random intercept for participant), revealed the main effects of ratio, *χ^2^* (1.00) = 55.32, *p* < 0.001, and congruity, *χ^2^* (2.00) = 168.14, *p* < 0.001, and a significant interaction between magnitude and congruity, *χ^2^* (2.00) = 6.15, *p* = 0.046 (all other *p*s > 0.18; see Figure 3). Post hoc comparisons indicated accuracy was higher for 2.00 ratio trials and, in descending order, congruent, neutral, and incongruent trials, with higher accuracy for number than cumulative area for neutral and incongruent trials (adj. *p*s < 0.01).

A linear mixed model with RT as the dependent variable and the same main effects and interactions as accuracy (random intercept for participant) revealed both similar and differential effects. There were main effects of magnitude, *F*(1.00, 9721.38) = 8.34, *p* = 0.004, Cohen’s *f* = 0.03, ratio, *F*(1.00, 9721.21) = 563.99, *p* < 0.001, Cohen’s *f* = 0.24, and congruity, *F*(2.00, 9721.25) = 51.55, *p* < 0.001, Cohen’s *f* = 0.10, and a significant interaction between ratio and congruity, *F*(2.00, 9721.18) = 11.45, *p* < 0.001, Cohen’s *f* = 0.05 (all other *p*s > 0.12; see Figure 3). In combination with RTs being longer for cumulative area than number and 1.25 than 2.00 ratio trials, post hoc analyses indicated that the interaction was driven by the ratio effect being significantly stronger for the neutral and congruent trials compared to the incongruent trials (adj. *p*s < 0.01; neutral vs. congruent *p* = 0.999).

Overall, the ratio and congruity effects observed in behavioral performance align with previous research: there was better performance with larger ratios and with congruent magnitude information.

### 3.2. ERP Results

**Figure 4:**
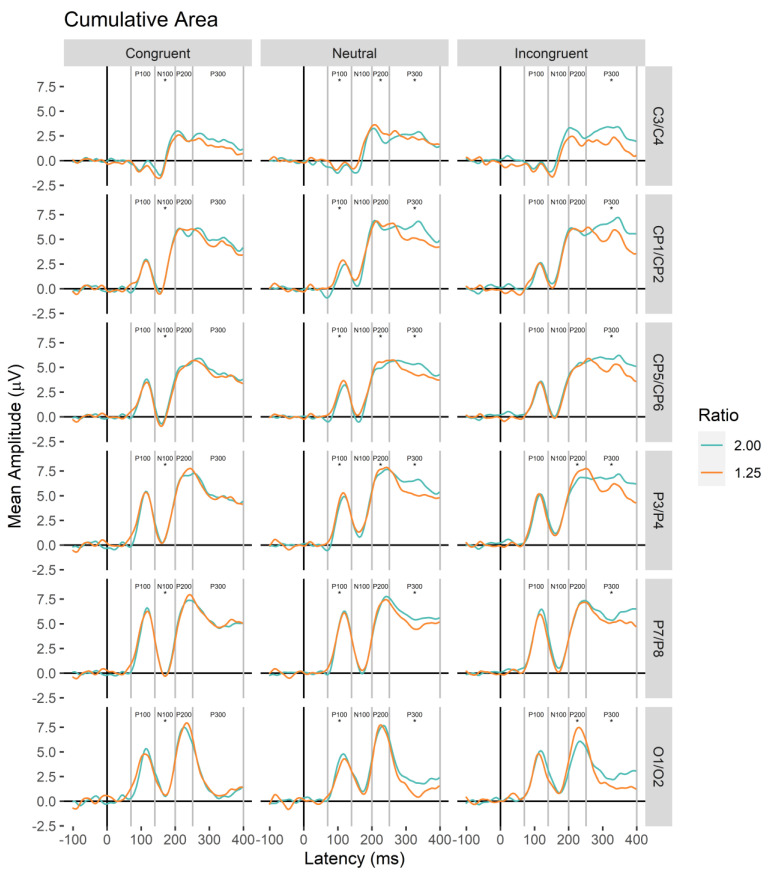
Cumulative area (CA) ERP waveform plots for each electrode pair (central, C3/C4, central-parietal CP1/CP2, lateral-central-parietal, CP5/CP6, parietal, P3/P4, lateral-parietal, P7/P8, occipital, O1/O2). Asterisks below each component label indicate whether significant ratio effects were observed in main or interaction effects in linear mixed models.

**Figure 5:**
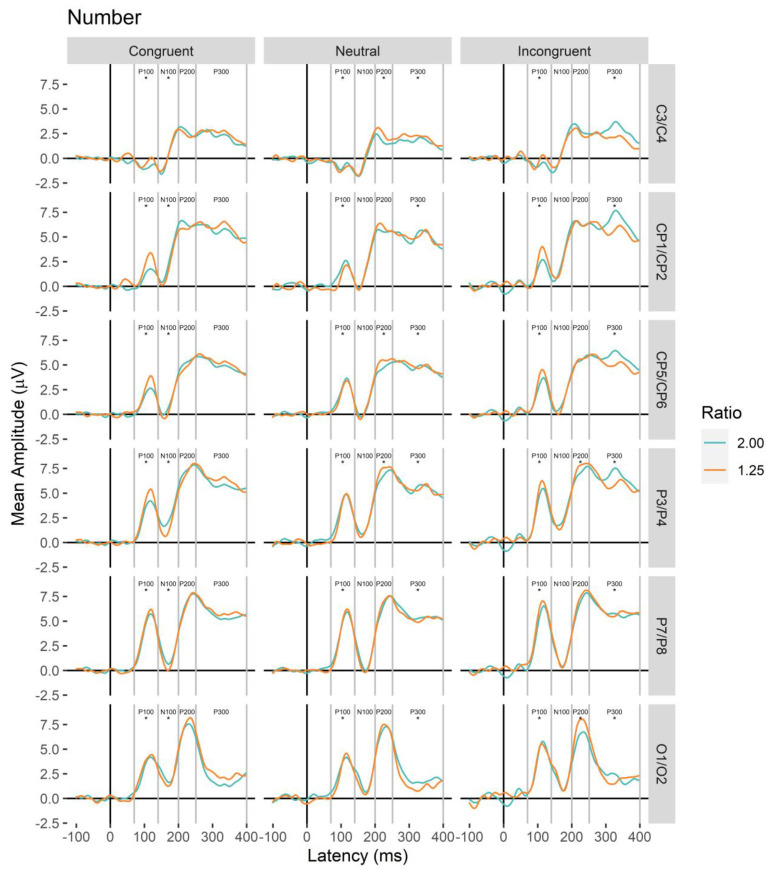
Non-symbolic number (Number) ERP waveform plots for each electrode pair (central, C3/C4, central-parietal CP1/CP2, lateral-central-parietal, CP5/CP6, parietal, P3/P4, lateral-parietal, P7/P8, occipital, O1/O2). Asterisks below each component label indicate whether significant ratio effects were observed in main or interaction effects in linear mixed models.

Analyses are arranged in the progression of neural processing starting with the onset of the stimulus (see Figure 4 and Figure 5 for electrode waveform plots). For each component, a linear mixed model predicted mean amplitude with main effects and interactions between magnitude, ratio (contrast coded: 1.25 Ratio = 1; 2.00 Ratio = −1), congruity, electrode pair (C3/C4, CP1/CP2, CP5/CP6, P3/P4, P7/P8, O1/O2), and electrode hemisphere (left, right; random intercept for participant). Effects that did not include the factors of interest (magnitude, ratio, congruity) are not reported here (see Supplementary Materials for these analyses).

### 3.3. P100

**Figure 6:**
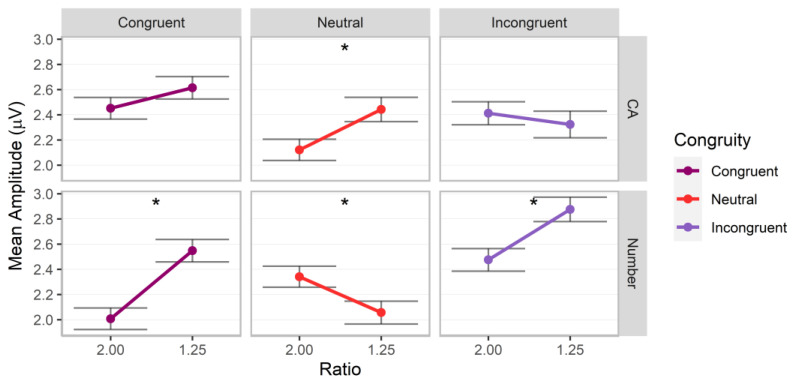
P100 mean amplitudes for post hoc comparisons investigating the interaction between magnitude (cumulative area = CA), ratio, and congruity. Error bars indicate standard errors. Asterisks indicate significant ratio effects (*p* < 0.05).

A significant interaction across magnitude, ratio, and congruity, *F*(2.00, 100,587.28) = 13.88, *p* < 0.001, Cohen’s *f* = 0.02, was observed (additional significant factor effects for ratio, congruity, ratio by congruity, magnitude by congruity, *p*s < 0.01; all other *p*s > 0.05). Post hoc analyses revealed that the interaction was driven by significant ratio effects, with greater values for the 1.25 ratio for cumulative area on neutral trials, coefficient = 0.18 (*SE* = 0.06), *z* = 2.89, adj. *p* = 0.004, and for number on congruent trials, coefficient 0.30 (*SE* = 0.06), *z* = 4.99, adj. *p* < 0.001, and incongruent trials, coefficient = 0.24 (*SE* = 0.06), *z* = 3.80, adj. *p* < 0.001, and a reverse ratio effect for number on neutral trials, −0.13 (*SE* = 0.06), *z* = −2.17, adj. *p* = 0.030 (other adj. *p*s > 0.05; Figure 6). For ratio effect coefficients by magnitude and congruity conditions, number was significantly stronger than cumulative area for congruent, difference estimate = −0.22 (*SE* = 0.09), *z* = −2.57, adj. *p* = 0.010, and incongruent trials, difference estimate = −0.29 (*SE* = 0.09), *z* = −3.11, adj. *p* = 0.002, and cumulative area was stronger than number for neutral trials, difference estimate = 0.32 (*SE* = 0.09), *z* = 3.59, adj. *p* < 0.001.

In sum, initial ratio effects were observed for both magnitudes in the P100 component, but these depended on congruity condition. The ratio effects differed in strength depending on participant judgments and were generally stronger for number than cumulative area.

### 3.4. N100

**Figure 7:**
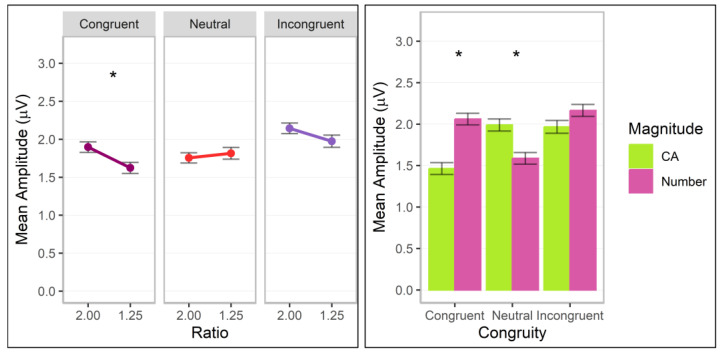
N100 mean amplitudes for post hoc comparisons investigating the interactions between congruity and ratio (**left** panel) and between magnitude (cumulative area = CA) and congruity (**right** panel). Error bars indicate standard errors. Asterisks indicate significant ratio effects (**left** panel) and magnitude differences (**right** panel; *p* < 0.05).

Significant interactions of ratio by congruity, *F*(2.00, 100585.07) = 23.75, *p* < 0.001, Cohen’s *f* = 0.02, and magnitude by congruity, *F*(2.00, 100585.07) = 23.75, *p* < 0.001, Cohen’s *f* = 0.02, were observed (additional significant factor effects for congruity, *p* < 0.001; all other *p*s > 0.05). The ratio by congruity interaction was driven by a significant ratio effect limited to the congruent condition with higher values for the 2.00 ratio, estimate = −0.12 (*SE* = 0.05) *z* = −2.50, adj. *p* = 0.012 (other adj. *p*s > 0.1; Figure 7, left panel). Post hoc analyses revealed that the magnitude by congruity interaction was driven by higher values for number compared to cumulative area for congruent trials, difference estimate = −0.56 (*SE* = 0.10), *z* = −5.87, adj. *p* < 0.001, with the reverse pattern observed for neutral trials, difference estimate = 0.39 (*SE* = 0.10), *z* = 3.90, adj. *p* < 0.001 (Figure 7, right panel).

In sum, ratio effects for the congruent condition were observed in the N100 component for both magnitudes.

### 3.5. P200

**Figure 8:**
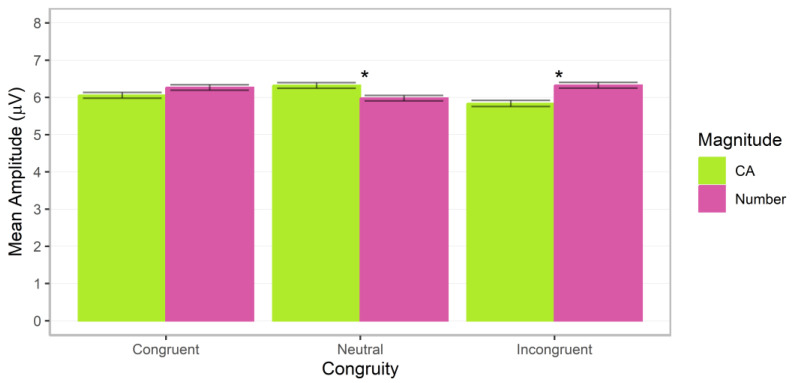
P200 mean amplitudes for post hoc comparisons investigating the interactions between magnitude (cumulative area = CA) by congruity. Error bars indicate standard errors. Asterisks indicate significant magnitude differences (*p* < 0.05).

**Figure 9:**
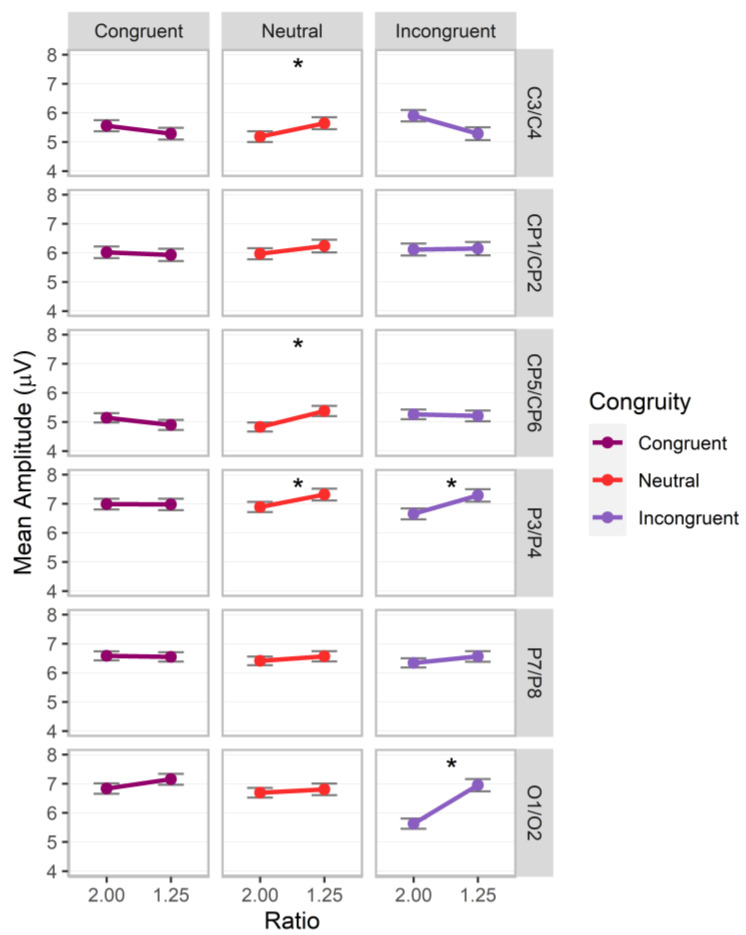
P200 mean amplitudes for post hoc comparisons investigating the interactions between electrode site (central, C3/C4, central-parietal CP1/CP2, lateral-central-parietal, CP5/CP6, parietal, P3/P4, lateral-parietal, P7/P8, occipital, O1/O2), ratio, and congruity. Error bars indicate standard errors. Asterisks indicate significant ratio effects (*p* < 0.05).

Significant interactions of magnitude by congruity, *F*(2.00, 100584.93) = 12.93, *p* < 0.001, Cohen’s *f* = 0.02, and ratio by congruity by pair, *F*(10.00, 100583.92) = 2.32, *p* = 0.010, Cohen’s *f* = 0.02, were observed (additional significant factor effects for ratio, ratio by congruity, ratio by pair, and congruity by pair, *p*s < 0.05; all other *p*s > 0.05). Post hoc analyses revealed that the magnitude by congruity interaction was driven by higher values for number compared to cumulative area for incongruent trials, with the reverse pattern observed for neutral trials (adj. *p*s < 0.05; see Figure 8). For electrode pairs, significant ratio effects with higher values for the 1.25 ratio were observed for neutral trials over C3/C4, estimate = 0.30 (*SE* = 0.13) *z* = 2.30, adj. *p* = 0.022, CP5/CP6, estimate = 0.33 (*SE* = 0.13) *z* = 2.57, adj. *p* = 0.010, and P3/P4, estimate = 0.27 (*SE* = 0.13) *z* = 2.09, adj. *p* = 0.037, and for incongruent trials over O1/O2, estimate = 0.71 (*SE* = 0.14) *z* = 5.22, adj. *p* < 0.001, and P3/P4, estimate = 0.36 (*SE* = 0.14) *z* = 2.70, adj. *p* = 0.007 (other adj. *p*s > 0.05; see Figure 9).

In sum, ratio effects contingent on congruity conditions continued into the P200 component for both magnitudes over central, parietal, and occipital sites.

### 3.6. P300

**Figure 10:**
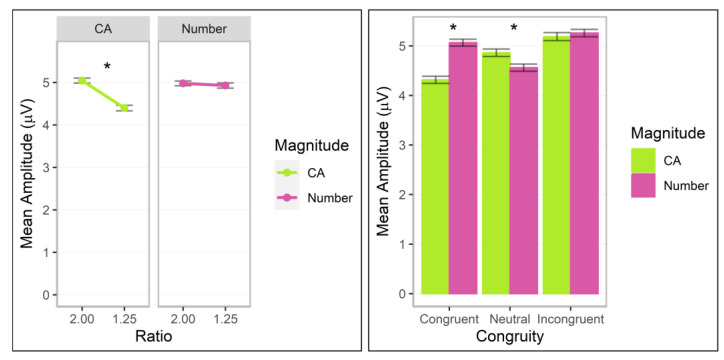
P300 mean amplitudes for post hoc comparisons investigating the interactions between magnitude (cumulative area = CA) and ratio (**left** panel) and between magnitude and congruity (**right** panel). Error bars indicate standard errors. Asterisks indicate significant ratio effects (**left** panel) and magnitude differences (**right** panel; *p* < 0.05).

**Figure 11:**
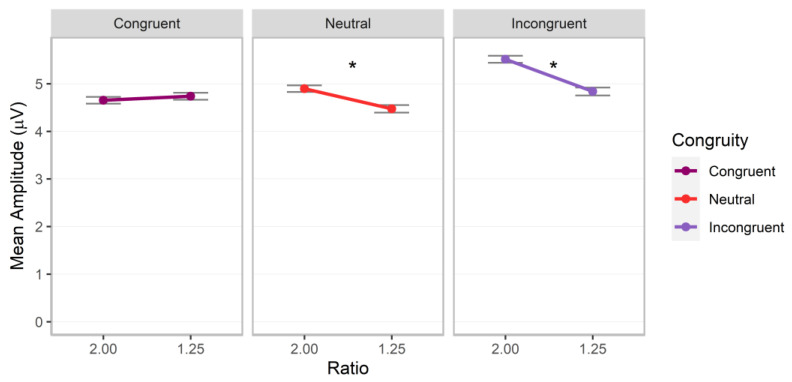
P300 mean amplitudes for post hoc comparisons investigating the interactions between ratio and congruity. Error bars indicate standard errors. Asterisks indicate significant ratio effects (*p* < 0.05).

Significant interactions between magnitude and ratio, *F*(1.00, 100585.08) = 23.99, *p* < 0.001, Cohen’s *f* = 0.02, magnitude and congruity, *F*(2.00, 100584.79) = 26.11, *p* < 0.001, Cohen’s *f* = 0.02, and ratio and congruity, *F*(2.00, 100585.66) = 11.55, *p* < 0.001, Cohen’s *f* = 0.02, were observed (additional factor effects of magnitude, ratio, congruity, *p*s < 0.01; all other *p*s > 0.10). Post hoc analyses indicated that the magnitude by ratio interaction was driven by a significant ratio effect, with higher values for the 2.00 ratio for cumulative area, estimate = −0.29 (*SE* = 0.04), *z* = −6.98, adj. *p* < 0.001, but not number, estimate = −0.01 (SE = 0.04), *z* = −0.20, adj. *p* = 0.839 (Figure 10, left panel). The ratio effect for cumulative area was significantly stronger than that for number, difference estimate = −0.28 (*SE* = 0.06), *z* = −4.90, adj. *p* < 0.001. The magnitude by congruity interaction was driven by higher values for cumulative area on congruent trials, estimate = 0.73 (*SE* = 0.10), *z* = 7.60, adj. *p* < 0.001, and number on neutral trials, estimate = 0.25 (*SE* = 0.10), *z* = 2.55, adj. *p* = 0.011 (Figure 10, right panel). The ratio by congruity interaction was driven by significant ratio effects, with higher values for 2.00 ratio trials for the neutral, estimate = −0.19 (*SE* = 0.05), *z* = −3.82, adj. *p* < 0.001, and incongruent conditions, estimate = −0.29 (*SE* = 0.05), *z* = −5.63, adj. *p* < 0.001, but no significant effect for congruent trials, estimate = 0.04 (*SE* = 0.05), *z* = 0.74, adj. *p* = 0.460 (see Figure 11).

In sum, ratio effects continued into the P300 component for cumulative area and number, and these were conditional on congruity for both magnitudes. Similar to the P100, ratio effects differed in strength depending on participant judgments; specifically, they were stronger for cumulative area than number.

### 3.7. ERP Results Summary

When surveying results across ERP waveforms, two main patterns emerged. First, ratio and congruity effects were present for both magnitudes in the P100 and continued through the P300, with some differences when participants judged number versus cumulative area. Second, when comparing the ratio effect across magnitudes, it was stronger for number in the early waveform (P100) and stronger for cumulative area in the later waveform (P300).

## 4. Discussion

The emergence of electrophysiological ratio and congruity effects as participants judged which of two stimulus arrays was greater in either number or cumulative area was examined in the present study. Initial ratio effects for number and cumulative area were observed in the P100. For number, this was largely consistent with previous research using non-symbolic stimuli, showing ratio effects in the C100 component [45,46] somewhat earlier than similar effects observed between 130 and 350 ms after stimulus onset over posterior sites [41,43,67,68]. For cumulative area, this was earlier than previous research observing ratio effects in the P300 [11] and similar in onset to prior observations in the N100 [44]. For both magnitude conditions (number and cumulative area), congruity effects were present during the initial ratio effects. This extends previous research in two important ways. First, it indicates that when judging number, while simultaneously varying cumulative area, a congruity effect is observed earlier than the P300 [11,13,69]. Second, it suggests congruity effects between cumulative area and number emerge in ERP waveforms when participants are required to explicitly judge cumulative area, extending previous research [11]. That the ratio effect for number in the P100 depended on congruity is in line with research indicating that numerosity differences can affect early waveforms, along with spatial magnitudes [46]. The results of the present study provide support for an early account of the congruity effect for non-symbolic number and cumulative area judgments.

### 4.1. Early Ratio and Congruity Effects for Magnitude Judgments

Initial ratio effects for both magnitudes appeared in the P100 and depended on congruity. The ratio effect for cumulative area was observed in the neutral condition whereas, for number, it was observed in the congruent and incongruent conditions, and with a different pattern in the neutral condition. Gebuis and colleagues (2010) reported P300 effects for number and size stimuli; the present findings extend these to the earlier P100 for cumulative area and number using non-symbolic stimuli. Overall, the present study provides novel evidence in favor of an early interaction account for cumulative area and number processing.

Importantly, evidence of early ratio and congruity effects further points to interacting perceptual processes when extracting numerical and cumulative area information. Prior ERP research has observed connections between the P100 component and perceptual processes including the processing of visual input [70] and orienting to spatial cues [71]. The ratio effects observed in the present study indicate that magnitude representations emerged by the P100. Furthermore, that the ratio effects depended on the congruity condition suggests that corresponding perceptual processes overlap, at least to some extent, for number and cumulative area. We propose that these early interactions reflect overlap in the mechanisms used to process cumulative area and number information. Similar to models of number perception [21], the detection and filtering of non-symbolic quantitative information may be shared across different magnitudes. This aligns with theories that basic visual characteristics are combined to generate numerical and spatial magnitude representations [72,73] or that numerical and non-numerical magnitudes are perceptually integral [74].

Nevertheless, there are open questions about why the interaction between ratio and congruity in the P100 differed for number and cumulative area. Although we can only speculate about possible answers, a possibility is that magnitudes vary in discriminability, affecting how perceptible differences in magnitude are to observers. Specifically, saliency differences between magnitudes may have been reflected in ERP waveforms in the present study. With regard to cumulative area, the ratio effects in the P100 were weaker, with differences between 1.25 and 2.00 ratio trials smaller than those for number. Moreover, whereas no significant interaction with magnitude was observed for the N100 or P200, the ratio effect was stronger for cumulative area compared to number in the P300. This may reflect a greater effort to attend to differences in spatial cues among more salient numerical information. The lower saliency for cumulative area information in the present stimuli may have initially elicited weaker corresponding representations and contributed to a more sustained neural response to support decision-making. This aligns with the increasing strength of the ratio effect with elapsed time for cumulative area in comparison to number.

### 4.2. Implications for Representing Number and Cumulative Area

Early ratio and congruity effects would seem to challenge the view that cumulative area is constructed from numerical representations. On this account, numerical information acts as an operator that transforms spatial information into cumulative area representations [23]. This necessitates that numerical representations be formed first, with subsequent manipulation of the spatial information so as to form a cumulative area representation. However, the cumulative area effects in the P100 would suggest that numerical information would need to be rapidly parsed, prior to the P100, after stimuli are presented. If not processed sequentially, a parallel account could allow for numerical information to function as a filter applied to spatial information to project cumulative area representations and vice versa for number representations. However, the presence of congruity effects challenges this view and it suggests that cumulative area influences number processing. Instead of an operator, or constructor role, for numerical information in cumulative area representation, the results of the present study are more parsimonious with a shared perceptual processing account.

Cumulative area processing may have interacted with the processing of non-symbolic number in the present study. This is suggested by the numerical ratio effect in the P100 being contingent on the congruity of cumulative area information. In addition, ratio and congruity effects were observed at similar electrode sites for both magnitudes. The role of spatial information, such as average size, convex hull, and density, in numerical representations has been previously proposed [72]. This is in line with the view that early ratio effects indicate common neural mechanisms for both types of magnitudes.

Yet differences in how ratio effects unfolded across time windows indicate that some processing differences between magnitudes may be present. Ratio effects for number were stronger than cumulative area in the P100 and weaker in the P300. This extends prior ERP research on non-symbolic number perception. Previous research has observed larger mean amplitudes for smaller than larger ratio trials in the P200 [41,42]. Numerical ratio effects, characterized by larger mean amplitudes for 1.25 than 2.00 ratio trials, for congruent and incongruent trials in the P100, as well as incongruent trials in the P200 at P3/P4 and O1/O2 sites, and neutral trials for C3/C4, CP5/CP6, and P3/P4 sites, align with this research. However, the ratio effect for neutral trials in the P100, congruent trials in the N100, and neutral and incongruent trials in the P300 displayed a reverse pattern. Although non-symbolic number research has not typically focused on later components, such as the P300, those that have, reported a similar effect of higher values for the greater ratio [43,75]. Shifts in ERP waveforms have also been reported for non-symbolic numbers across the earlier C1 and P200 time windows [46]. In the present study, these differences may be attributable to the inclusion of explicit cumulative area judgments. In line with ERP research examining conflict resolution when preparing motor responses [76], the change in direction of the ratio effect may be a result of resolving which array is greater in the focal magnitude when multiple magnitudes are available. Overall, the ratio and congruity effects in the P100 and P300 in the present study are in line with overlapping perceptual processes for forming and sustaining number and cumulative area representations.

### 4.3. Limitations and Future Directions

There are a few limitations in the design of the present study. First, although we focused on cumulative area specifically, a remaining question is whether cumulative area was indeed the type of spatial information that was driving the observed effects. As detailed in previous research, multiple types of spatial information are correlated with cumulative area when presented via arrays of visual objects including spacing, average size, and perimeter [77,78]. Furthermore, questions have been raised regarding whether mental representations of cumulative area are based on mathematical calculations of size (c.f. [78,79]). Prior research suggests that numerical information may be relatively more salient than other magnitudes [15,23], at least when comparing stimuli matched in mathematical ratio rather than perceptual discriminability [74,80]. However, evidence from behavioral studies suggests that cumulative area in particular interacts with number [80,81] compared to other spatial magnitudes. In the present study, stimuli were created using mathematical cumulative area, and some controls were used to reduce other spatial parameters as cues. However, since some information covaried with cumulative area, we cannot rule out whether other spatial magnitudes account for cumulative area congruity effects in the present study. Controlling for several types of correlated spatial information might allow future studies to examine whether early congruity effects, when making non-symbolic number comparisons, are specific to cumulative area.

We have emphasized the overlap between number and cumulative area; this may extend to other types of magnitude. Others have suggested possible interactions with number and other magnitudes, such as density and convex hull [72]. By applying advanced models of stimulus generation procedures [77], future research may be in a position to systematically manipulate multiple spatial magnitudes to identify the relative contributions of each to early emerging ratio effects in ERP waveforms. Subsequent studies might also consider the use of exploratory approaches to identify electrode sites and time windows when comparing other magnitudes using ERPs. Using multiple comparison testing to identify scalp-wide time windows of interest (e.g., [82,83]) can further detect whether differences are present when comparing the onset of such effects across magnitudes. Future research would do well to consider the timing of ratio and congruity effects for different magnitudes to examine whether these are represented in the visual system with overlapping or distinct processes.

In addition, we would urge future studies to consider manipulating instructions to examine top-down effects on the emergence of ratio and congruity effects. Although we have emphasized the potential overlap in perceptual processing, it is unclear to what extent the overlap is influenced by top-down attentional mechanisms, particularly when a task explicitly labels the target dimension. In Gebuis and Reynvoet (2012), differences in ERP waveforms were observed for numerical and spatial magnitudes when participants were, and were not, explicitly told to attend to such cues [67]. Using a similar method, studies might test whether the onset of ratio and congruity effects in ERP waveforms vary when participants’ instructions to explicitly attend to number and cumulative area are manipulated.

The early onset of ratio and congruity effects suggests that corresponding ERP components could be used as indicators of magnitude processing ability. Prior research has observed correlations between number-related effects in ERP components and behavioral performance. For example, the P200 amplitude was found to vary in children with lower versus higher math ability [84] and behavioral performance when comparing non-symbolic number arrays and measures of probabilistic reasoning were associated with distance effects in the N300 amplitude [85]. Future research might investigate whether ratio and congruity effects for cumulative area and number in the P100 are predictive of performance on mathematical tasks, so as to better understand the potential links between magnitude perception and higher-level cognitive processing.

## 5. Conclusions

The emergence of neural ratio and congruity effects were investigated when participants made magnitude judgments about cumulative area and non-symbolic number using ERPs. Initial ratio and congruity effects, including interactions between ratio and congruity, for cumulative area and number emerged in the P100. This evidence provides sup-port for an early account of the interactions between cumulative area and number processing. Furthermore, the presence of congruity and ratio effects in the P100 suggests, at least partially, shared perceptual mechanisms for forming magnitude representations of cumulative area and number. These results contribute to models of magnitude representation, with evidence that interactions between number and cumulative area may occur early in processing. 

## Data Availability

The data that support the findings of this study are openly available in Open Science Framework at https://doi.org/10.17605/OSF.IO/RJ7US.

## Supplementary Materials

The following supporting information can be downloaded at: https://www.mdpi.com/article/10.3390/brainsci13060975/s1, Table S1: Specific cumulative area (CA), number, and average size (Avg. Size) dimensions of stimuli in each trial type; Table S2: Main effects and interactions observed in linear mixed model predicting mean amplitude; Table S3: Main effects and interactions observed in linear mixed model predicting mean amplitude; Table S4: Main effects and interactions observed in linear mixed model predicting mean amplitude; Table S5: Main effects and interactions observed in linear mixed model predicting mean amplitude
