# Supplementary material for: Electrophysiological Comparison of Cumulative Area and Non-Symbolic Number Judgments
Source: Brain Sci. 2023 Jun 20;13(6):975. doi: 10.3390/brainsci13060975 (PMC10296489; doi:10.3390/brainsci13060975)
Supplement: Supplementary file 1 [file brainsci-13-00975-s001.zip › brainsci-2432394-supplementary.pdf]

Stimuli Specifications

Table S1

Specific cumulative area (CA), number, and average size (Avg. Size) dimensions of stimuli in each trial type

	Trial Type	Number, 2.00 Ratio, Congruent	Number, 2.00 Ratio, Neutral	Number, 2.00 Ratio, Incongruent	Number, 1.25 Ratio, Congruent	Number, 1.25 Ratio, Neutral	Number, 1.25 Ratio, Incongruent
Stimulus Greater in Target Magnitude	CA (cm^2)	20.6	15.45	10.3	17.2	15.45	13.7
	Number	24	24	24	20	20	20
	Avg. Size (cm^2)	0.86	0.64	0.43	0.86	0.77	0.69
Stimulus Lesser in Target Magnitude	CA (cm^2)	10.3	15.45	20.6	13.7	15.45	17.2
	Number	12	12	12	16	16	16
	Avg. Size (cm^2)	0.86	1.29	1.72	0.86	0.97	1.08

	Trial Type	CA, 2.00 Ratio, Congruent	CA, 2.00 Ratio, Neutral	CA, 2.00 Ratio, Incongruent	CA, 1.25 Ratio, Congruent	CA, 1.25 Ratio, Neutral	CA, 1.25 Ratio, Incongruent
Stimulus Greater in Target Magnitude	CA (cm^2)	20.6	20.6	20.6	17.2	17.2	17.2
	Number	24	18	12	20	18	16
	Avg. Size (cm^2)	0.86	1.14	1.72	0.86	0.96	1.08
Stimulus Lesser in Target Magnitude	CA (cm^2)	10.3	10.3	10.3	13.7	13.7	13.7
	Number	12	18	24	16	18	20
	Avg. Size (cm^2)	0.86	0.57	0.43	0.86	0.76	0.69

ERP Waveform Results

P100

Table S2

Main effects and interactions observed in linear mixed model predicting mean amplitude.

Effect	Sum Sq	Mean Sq	NumDF	DenDF	F value	Pr(>F)	cohensf
Magnitude	0.70	0.70	1.00	100587.74	0.01	0.917	0.000
Ratio	1060.91	1060.91	1.00	100590.69	16.34	0.000	0.013
Congruity	1355.22	677.61	2.00	100586.44	10.43	0.000	0.014
pair	113421.80	22684.36	5.00	100584.10	349.28	0.000	0.132
hemi	2338.91	2338.91	1.00	100584.10	36.01	0.000	0.019
Magnitude:Ratio	100.69	100.69	1.00	100586.90	1.55	0.213	0.004
Magnitude:Congruity	1295.33	647.67	2.00	100586.13	9.97	0.000	0.014
Ratio:Congruity	469.05	234.52	2.00	100588.25	3.61	0.027	0.008
Magnitude:pair	37.13	7.43	5.00	100584.10	0.11	0.989	0.002
Ratio:pair	227.53	45.51	5.00	100584.10	0.70	0.623	0.006
Congruity:pair	234.75	23.47	10.00	100584.10	0.36	0.963	0.006
Magnitude:hemi	28.38	28.38	1.00	100584.10	0.44	0.509	0.002
Ratio:hemi	13.67	13.67	1.00	100584.10	0.21	0.646	0.001
Congruity:hemi	220.38	110.19	2.00	100584.10	1.70	0.183	0.006
pair:hemi	13617.79	2723.56	5.00	100584.10	41.94	0.000	0.046
Magnitude:Ratio:Congruity	1803.21	901.60	2.00	100587.28	13.88	0.000	0.017
Magnitude:Ratio:pair	54.41	10.88	5.00	100584.10	0.17	0.975	0.003
Magnitude:Congruity:pair	219.86	21.99	10.00	100584.10	0.34	0.971	0.006
Ratio:Congruity:pair	136.43	13.64	10.00	100584.10	0.21	0.996	0.005
Magnitude:Ratio:hemi	6.35	6.35	1.00	100584.10	0.10	0.755	0.001
Magnitude:Congruity:hemi	90.83	45.42	2.00	100584.10	0.70	0.497	0.004
Ratio:Congruity:hemi	46.64	23.32	2.00	100584.10	0.36	0.698	0.003
Magnitude:pair:hemi	68.27	13.65	5.00	100584.10	0.21	0.958	0.003
Ratio:pair:hemi	13.49	2.70	5.00	100584.10	0.04	0.999	0.001
Congruity:pair:hemi	139.37	13.94	10.00	100584.10	0.21	0.995	0.005
Magnitude:Ratio:Congruity:pair	694.52	69.45	10.00	100584.10	1.07	0.382	0.010
Magnitude:Ratio:Congruity:hemi	36.09	18.04	2.00	100584.10	0.28	0.757	0.002
Magnitude:Ratio:pair:hemi	10.29	2.06	5.00	100584.10	0.03	0.999	0.001
Magnitude:Congruity:pair:hemi	80.13	8.01	10.00	100584.10	0.12	1.000	0.004
Ratio:Congruity:pair:hemi	20.83	2.08	10.00	100584.10	0.03	1.000	0.002
Magnitude:Ratio:Congruity:pair:hemi	66.77	6.68	10.00	100584.10	0.10	1.000	0.003

N100

Table S3

Main effects and interactions observed in linear mixed model predicting mean amplitude.

Effect	Sum Sq	Mean Sq	NumDF	DenDF	F value	Pr(>F)	cohensf
Magnitude	281.68	281.68	1.00	100585.96	3.40	0.065	0.006
Ratio	74.28	74.28	1.00	100587.60	0.90	0.344	0.003
Congruity	1900.27	950.14	2.00	100585.23	11.46	0.000	0.015
pair	20018.90	4003.78	5.00	100583.98	48.28	0.000	0.049
hemi	334.81	334.81	1.00	100583.98	4.04	0.045	0.006
Magnitude:Ratio	16.00	16.00	1.00	100585.49	0.19	0.661	0.001
Magnitude:Congruity	3938.73	1969.36	2.00	100585.07	23.75	0.000	0.022
Ratio:Congruity	629.99	314.99	2.00	100586.28	3.80	0.022	0.009
Magnitude:pair	244.29	48.86	5.00	100583.98	0.59	0.708	0.005
Ratio:pair	44.37	8.87	5.00	100583.98	0.11	0.991	0.002
Congruity:pair	817.31	81.73	10.00	100583.98	0.99	0.453	0.010
Magnitude:hemi	13.38	13.38	1.00	100583.98	0.16	0.688	0.001
Ratio:hemi	15.79	15.79	1.00	100583.98	0.19	0.663	0.001
Congruity:hemi	12.72	6.36	2.00	100583.98	0.08	0.926	0.001
pair:hemi	1466.64	293.33	5.00	100583.98	3.54	0.003	0.013
Magnitude:Ratio:Congruity	319.40	159.70	2.00	100585.72	1.93	0.146	0.006
Magnitude:Ratio:pair	78.52	15.70	5.00	100583.98	0.19	0.967	0.003
Magnitude:Congruity:pair	461.46	46.15	10.00	100583.98	0.56	0.850	0.007
Ratio:Congruity:pair	723.42	72.34	10.00	100583.98	0.87	0.559	0.009
Magnitude:Ratio:hemi	2.12	2.12	1.00	100583.98	0.03	0.873	0.001
Magnitude:Congruity:hemi	13.66	6.83	2.00	100583.98	0.08	0.921	0.001
Ratio:Congruity:hemi	75.10	37.55	2.00	100583.98	0.45	0.636	0.003
Magnitude:pair:hemi	13.71	2.74	5.00	100583.98	0.03	0.999	0.001
Ratio:pair:hemi	21.39	4.28	5.00	100583.98	0.05	0.998	0.002
Congruity:pair:hemi	102.30	10.23	10.00	100583.98	0.12	1.000	0.004
Magnitude:Ratio:Congruity:pair	227.17	22.72	10.00	100583.98	0.27	0.987	0.005
Magnitude:Ratio:Congruity:hemi	83.94	41.97	2.00	100583.98	0.51	0.603	0.003
Magnitude:Ratio:pair:hemi	18.08	3.62	5.00	100583.98	0.04	0.999	0.001
Magnitude:Congruity:pair:hemi	84.40	8.44	10.00	100583.98	0.10	1.000	0.003
Ratio:Congruity:pair:hemi	31.79	3.18	10.00	100583.98	0.04	1.000	0.002
Magnitude:Ratio:Congruity:pair:hemi	41.94	4.19	10.00	100583.98	0.05	1.000	0.002

P200

Table S4

Main effects and interactions observed in linear mixed model predicting mean amplitude.

Effect	Sum Sq	Mean Sq	NumDF	DenDF	F value	Pr(>F)	cohensf
Magnitude	43.46	43.46	1.00	100585.76	0.47	0.492	0.002
Ratio	1480.68	1480.68	1.00	100587.29	16.10	0.000	0.013
Congruity	63.31	31.65	2.00	100585.08	0.34	0.709	0.003
pair	43850.66	8770.13	5.00	100583.92	95.35	0.000	0.069
hemi	1212.64	1212.64	1.00	100583.92	13.18	0.000	0.011
Magnitude:Ratio	123.94	123.94	1.00	100585.32	1.35	0.246	0.004
Magnitude:Congruity	2377.81	1188.91	2.00	100584.93	12.93	0.000	0.016
Ratio:Congruity	1235.76	617.88	2.00	100586.07	6.72	0.001	0.012
Magnitude:pair	279.45	55.89	5.00	100583.92	0.61	0.694	0.005
Ratio:pair	1309.22	261.84	5.00	100583.92	2.85	0.014	0.012
Congruity:pair	1756.51	175.65	10.00	100583.92	1.91	0.039	0.014
Magnitude:hemi	0.01	0.01	1.00	100583.92	0.00	0.990	0.000
Ratio:hemi	7.95	7.95	1.00	100583.92	0.09	0.769	0.001
Congruity:hemi	7.35	3.67	2.00	100583.92	0.04	0.961	0.001
pair:hemi	5272.49	1054.50	5.00	100583.92	11.46	0.000	0.024
Magnitude:Ratio:Congruity	99.66	49.83	2.00	100585.54	0.54	0.582	0.003
Magnitude:Ratio:pair	107.79	21.56	5.00	100583.92	0.23	0.948	0.003
Magnitude:Congruity:pair	442.82	44.28	10.00	100583.92	0.48	0.903	0.007
Ratio:Congruity:pair	2130.41	213.04	10.00	100583.92	2.32	0.010	0.015
Magnitude:Ratio:hemi	223.23	223.23	1.00	100583.92	2.43	0.119	0.005
Magnitude:Congruity:hemi	68.89	34.44	2.00	100583.92	0.37	0.688	0.003
Ratio:Congruity:hemi	78.79	39.39	2.00	100583.92	0.43	0.652	0.003
Magnitude:pair:hemi	17.29	3.46	5.00	100583.92	0.04	0.999	0.001
Ratio:pair:hemi	20.30	4.06	5.00	100583.92	0.04	0.999	0.001
Congruity:pair:hemi	89.13	8.91	10.00	100583.92	0.10	1.000	0.003
Magnitude:Ratio:Congruity:pair	196.73	19.67	10.00	100583.92	0.21	0.995	0.005
Magnitude:Ratio:Congruity:hemi	135.67	67.84	2.00	100583.92	0.74	0.478	0.004
Magnitude:Ratio:pair:hemi	47.06	9.41	5.00	100583.92	0.10	0.992	0.002
Magnitude:Congruity:pair:hemi	58.18	5.82	10.00	100583.92	0.06	1.000	0.003
Ratio:Congruity:pair:hemi	40.67	4.07	10.00	100583.92	0.04	1.000	0.002
Magnitude:Ratio:Congruity:pair:hemi	71.72	7.17	10.00	100583.92	0.08	1.000	0.003

P300

Table S5

Main effects and interactions observed in linear mixed model predicting mean amplitude.

Effect	Sum Sq	Mean Sq	NumDF	DenDF	F value	Pr(>F)	cohensf
Magnitude	886.36	886.36	1.00	100585.42	10.76	0.001	0.010
Ratio	2206.33	2206.33	1.00	100586.59	26.78	0.000	0.016
Congruity	5214.99	2607.49	2.00	100584.90	31.65	0.000	0.025
pair	152745.12	30549.02	5.00	100584.02	370.87	0.000	0.136
hemi	630.01	630.01	1.00	100584.02	7.65	0.006	0.009
Magnitude:Ratio	1975.95	1975.95	1.00	100585.08	23.99	0.000	0.015
Magnitude:Congruity	4301.13	2150.57	2.00	100584.79	26.11	0.000	0.023
Ratio:Congruity	1903.01	951.50	2.00	100585.66	11.55	0.000	0.015
Magnitude:pair	36.52	7.30	5.00	100584.02	0.09	0.994	0.002
Ratio:pair	272.97	54.59	5.00	100584.02	0.66	0.652	0.006
Congruity:pair	203.34	20.33	10.00	100584.02	0.25	0.991	0.005
Magnitude:hemi	65.25	65.25	1.00	100584.02	0.79	0.373	0.003
Ratio:hemi	0.00	0.00	1.00	100584.02	0.00	0.998	0.000
Congruity:hemi	7.50	3.75	2.00	100584.02	0.05	0.955	0.001
pair:hemi	9382.26	1876.45	5.00	100584.02	22.78	0.000	0.034
Magnitude:Ratio:Congruity	85.50	42.75	2.00	100585.26	0.52	0.595	0.003
Magnitude:Ratio:pair	29.41	5.88	5.00	100584.02	0.07	0.996	0.002
Magnitude:Congruity:pair	759.81	75.98	10.00	100584.02	0.92	0.511	0.010
Ratio:Congruity:pair	816.10	81.61	10.00	100584.02	0.99	0.449	0.010
Magnitude:Ratio:hemi	0.94	0.94	1.00	100584.02	0.01	0.915	0.000
Magnitude:Congruity:hemi	6.69	3.35	2.00	100584.02	0.04	0.960	0.001
Ratio:Congruity:hemi	2.13	1.06	2.00	100584.02	0.01	0.987	0.001
Magnitude:pair:hemi	35.50	7.10	5.00	100584.02	0.09	0.994	0.002
Ratio:pair:hemi	57.44	11.49	5.00	100584.02	0.14	0.983	0.003
Congruity:pair:hemi	13.12	1.31	10.00	100584.02	0.02	1.000	0.001
Magnitude:Ratio:Congruity:pair	224.52	22.45	10.00	100584.02	0.27	0.987	0.005
Magnitude:Ratio:Congruity:hemi	330.37	165.18	2.00	100584.02	2.01	0.135	0.006
Magnitude:Ratio:pair:hemi	34.22	6.84	5.00	100584.02	0.08	0.995	0.002
Magnitude:Congruity:pair:hemi	155.46	15.55	10.00	100584.02	0.19	0.997	0.004
Ratio:Congruity:pair:hemi	64.52	6.45	10.00	100584.02	0.08	1.000	0.003
Magnitude:Ratio:Congruity:pair:hemi	39.71	3.97	10.00	100584.02	0.05	1.000	0.002
